# Reconstruction of a surgical defect in the popliteal fossa: A case report

**DOI:** 10.1016/j.ijscr.2018.10.070

**Published:** 2018-11-01

**Authors:** Dan C. Filitis, Juliya Fisher, Faramarz H. Samie

**Affiliations:** Columbia University Medical Center, Department of Dermatology, New York, NY, 10032, United States

**Keywords:** Case report, Popliteal fossa reconstruction, Bilobed flap, Porcine xenograft, Mohs micrographic surgery

## Abstract

**Introduction:**

Surgical defects of the popliteal fossa pose a reconstructive challenge to the surgeon, given the intimate relationship of this area with the knee joint and underlying vital neuro-vasculature.

**Case presentation:**

An 88-year-old woman was treated for a biopsy proven invasive squamous cell carcinoma of the right popliteal fossa with Mohs micrographic surgery. The resultant defect (4.5 × 4.3 cm) was repaired using a random patterned bilobed flap with an undersized secondary lobe and porcine xenograft with excellent functional and cosmetic outcome.

**Discussion:**

Numerous approaches to reconstruction of the popliteal fossa have been proposed, including primary closure, secondary intent healing, skin grafting, random flaps, muscle flaps, fasciocutaneous flaps, freeflaps, and tissue expansion. Sub-optimal repair of the popliteal fossa can reduce mobility of the knee joint, carry a high risk of wound dehiscence and may result in significant scarring. Here, we present the use of a bilobed flap with an undersized secondary lobe, in a patient with limited tissue laxity, for the repair of the popliteal fossa.

**Conclusion:**

Utilizing a random pattern bilobed flap with an undersized secondary lobe is an excellent alternative for the repair of moderate sized popliteal fossa defects.

## Highlights

• Popliteal tumors and their associated defects present the dermatologic surgeon with rare and unique surgical and reconstruction challenges.
• When planning reconstruction in the popliteal fossa, prolonged wound care and functional impairment are concerns.
• In such an area with little tissue laxity, a random patterned bilobed with an undersized secondary lobe is an excellent solution to this challenge.

## 1 Introduction

Cutaneous malignancies at the popliteal fossa are not common, however, the resultant post-extirpation defects can be challenging to reconstruct. This region is intimately involved with mobility of a large articulation – the knee – and overlies with proximity, vital lower-extremity vessels. Furthermore, the fossa’s location on the lower extremity predisposes procedures at the site to both prolonged healing, secondary to decreased perfusion and a higher infection risk. Considering the aforementioned, a successful and reproducible surgical approach must consider anatomic and functional aspects alongside cosmesis.

The authors report a case of a moderately sized Mohs micrographic surgery defect in the right popliteal fossa and subsequent repair, with excellent restoration of function and cosmesis.

This work is reported in line with the SCARE criteria [1].

## 2 Presentation of case

**Fig. 1:**
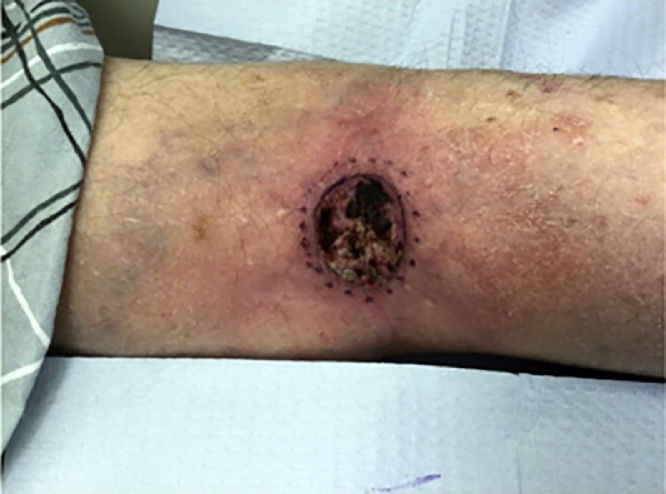
Tumor - Biopsy-proven invasive squamous cell carcinoma of the right popliteal fossa measuring 3.9 × 3.7 cm.

**Fig. 2:**
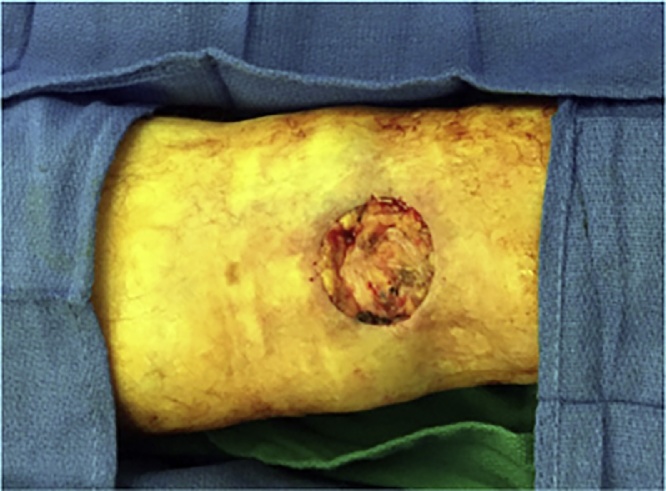
Defect - 4.5 × 4.3 cm final Mohs defect after extirpation of invasive squamous cell carcinoma on the right popliteal fossa.

**Fig. 3:**
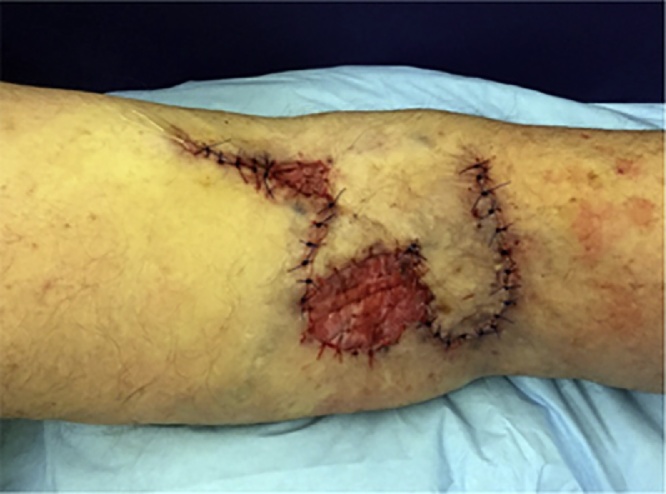
Repair - Combination lateral-based bilobed flap and porcine xenograft repair of post-extirpation defect after Mohs of invasive squamous cell carcinoma on the right popliteal fossa - as seen immediately after reconstruction.

**Fig. 4:**
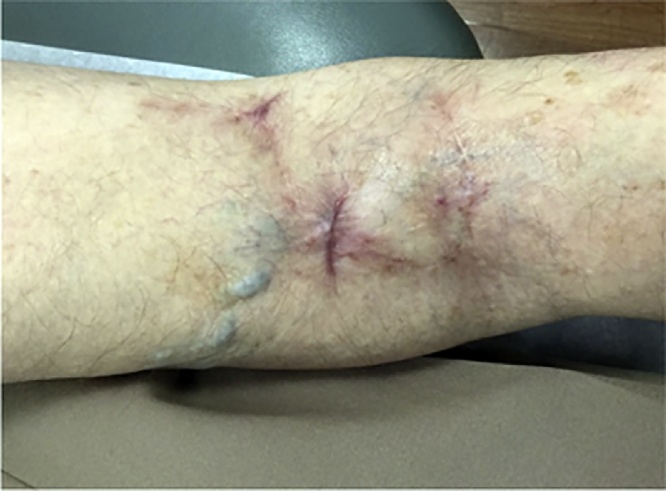
3-month follow-up - Appearance of combination lateral-based bilobed flap and porcine xenograft repair of the right popliteal fossa - as seen at 3-month follow-up.

An 88-year-old woman presented with a biopsy-proven invasive squamous cell carcinoma of the right popliteal fossa (Fig. 1). The tumor was cleared after one stage of Mohs micrographic surgery with a final surgical defect measuring 4.5 × 4.3 cm (Fig. 2). The wound was repaired with a combination random-pattern bilobed transposition flap with undersized secondary lobe and porcine xenograft. A lateral-based bilobed transposition flap was designed proximally and adjacent to the defect. The flap borders, including the primary and secondary lobes, and a standing cone at the advancing edge of the primary lobe, were incised down to the subcutaneous fat. The flap was then elevated and standing cone was removed. The flap was transposed – the primary lobe to fill the primary defect and the secondary lobe to resurface a portion of the defect resulting from the movement of the primary lobe. The remainder of the defect from the transposition of the primary lobe was repaired with a porcine xenograft (Fig. 3). A xenograft was utilized because the secondary lobe was relatively undersized due to lack of sufficient tissue laxity. Here, the bilobed flap was not large enough to cover the primary defect and the entirety of the defect resulting from the transposition of the primary lobe. Nevertheless, the flap allowed for the displacement of a wound from the popliteal fossa to the infero-posterior thigh, which was both smaller and positioned in a more advantageous location for second intention healing. The xenograft served as a biologic dressing and possibly decreased the immediate risk of postoperative bleeding as it was sutured in place. At 3-month follow-up, the patient had excellent cosmetic and functional results (Fig. 4).

## 3 Discussion

While there are few publications on reconstruction of the popliteal fossa, mostly in the setting of trauma and burn scars, various techniques have been described and may be employed as dictated by the resultant defect’s characteristics and surgeon preference. The predictability of scar outcome and cosmesis makes primary closure favorable whenever possible, but in the popliteal region the approach is typically not suitable for larger defects due to potential restriction of joint mobility and higher risk of dehiscence. Secondary intention healing is also not an ideal choice for flexion creases at large joints as they can result in contracture that precludes full limb movement, as well as prolonged healing and conspicuous unsightly scarring. Skin grafts reduce the possibility of contracture, however, they require extended immobilization with splinting and meticulous dressing changes at the expense of prolonged patient discomfort. Despite their limitations, primary closure, granulation, and grafts join a host of previously described approaches that have been successfully used to repair defects in the popliteal fossa, including – random flaps, muscle flaps, fasciocutaneous flaps, free-flaps, and tissue expansion. [2]

Since it’s description in 1918, the bilobed flap has been a workhorse for nasal reconstruction, and through a series of modifications, has become recognized as an adaptable and dependable reconstructive option at anatomic sites beyond the nose, including the popliteal fossa. For larger defects, an axial-based approach to the bilobed, allowing for larger flap dimensions and enhanced viability, has been described using the sural arteries [3]. However, when considering smaller defects, a random-pattern design can also be successfully executed and may be a better alternative [4]. The latter avoids axial-associated challenges, including – issues with the donor site, prolonged surgery times, and increased technical challenges – and when defect-dimensions allow, may be favored over muscle flaps, fasciocutaneous flaps, free flaps, and tissues expansion. Furthermore, as demonstrated herein, for moderately sized defects, even when down-sized for the respective defect, the bilobed-flap can significantly decrease defect size and strategically displace the portion of the wound that would heal by second intention from the popliteal fossa to the infero-posterior thigh with minimal impact on the postoperative course. Given that the popliteal fossa overlies a joint, one of the goals in our case was to minimize scarring in this area. We achieved this by using the primary lobe of our bilobed flap to cover the wound overlying the joint and displacing the secondary defect to a less significant location. The resultant wound was covered by a porcine xenograft given that this smaller defect was not in an area important for functionality.

As with all cutaneous reconstructions, complications associated with bilobed flaps such as hemorrhage, hematoma formation, infection, wound dehiscence, and trap-dooring are all possible. Furthermore, healing by second intention over a joint, may impair mobility and prolong healing. However, with proper patient selection, appropriate flap design, and meticulous surgical execution, all complications mentioned can be minimized or altogether avoided. No complications were observed in our case.

## 4 Conclusions

Popliteal tumors and associated post-extirpation defects present the dermatologic surgeon with rare and unique surgical and reconstruction challenges that demand both functional and cosmetic considerations in the setting of lower-extremity-associated perfusion and infection risk. As such, a thorough understanding of anatomy, reconstructive options, and associated complications are crucial for reproducible high-quality patient care. Popliteal fossa reconstruction with a random-pattern based bilobed flap is a favorable alternative to technically cumbersome and time-consuming axial, free-flap, or tissue expansion approaches for small to moderately sized defects in the region. As demonstrated in this case, bilobed flap with an undersized secondary lobe can be successfully applied to the reconstruction of the popliteal fossa with minimal impact on joint function and cosmetic outcome while minimizing prolonged joint immobility that is so often a concern with reconstructions at articulations.

## Conflicts of interest

Dan C. Filitis, Juliya Fisher and Faramarz H. Samie have no conflicts of interest to disclose.

## Funding

This research is not funded.

## Ethical approval

This case report does not require ethical approval.

## Consent

A written consent was obtained and is available upon request.

## Author contribution

Dan C. Filitis: case report design, writing, subject research, care of patient.

Juliya Fisher: case report design, writing, subject research.

Faramarz H. Samie: case report design, care of patient, subject research, review and final approval of manuscript.

## Registration of research studies

None.

## Guarantor

Faramarz H. Samie.

## Provenance and peer review

Not commissioned, externally peer reviewed.
